# The Exception That Proves the Rule: An Interview with Jenny Graves

**DOI:** 10.1371/journal.pgen.1000063

**Published:** 2008-06-27

**Authors:** Jane Gitschier

**Affiliations:** Departments of Medicine and Pediatrics, Institute for Human Genetics, University of California San Francisco, San Francisco, California, United States of America

**Image 1:**
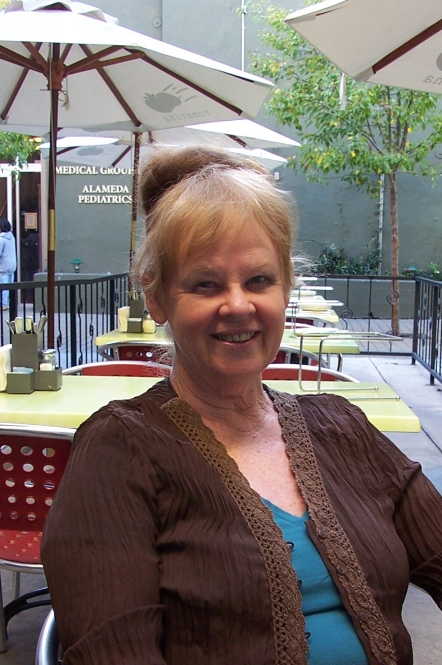
Jenny Graves

Close to 20 years ago, I was contacted by an Australian woman who was planning to map the locations of genes that are X-linked in humans in some odd Australian critters, the monotremes. These animals comprise a distantly related branch of mammals that have hair and lactate, but additionally lay eggs. She wanted a probe from our lab, and, in exchange, little vials of DNA from spiny echidna and platypus appeared in the mail. Our lab became enamoured of these singular animals, and we followed their scientific story with great interest. The lady was Jenny Graves [Image 1], and it has taken me this long to finally meet her.

Jenny is a native Australian, and one of her first dips into research as an undergraduate honours student involved asking whether kangaroos employ X-inactivation [they do]. Although she deviated from this interest as a PhD student in Berkeley and fully expected to follow a different research path, she found herself back in Australia, post-PhD, where a colleague suggested she look at gene mapping in marsupials. Though her curiosity about X-inactivation has been the driving force for much of her work, this serendipitous suggestion proved pivotal. Jenny's work on monotremes and marsupials has led to powerful insights into the evolution and function of the X and Y chromosomes in mammals.

Jenny has had a long and joyful career, overcoming a near fatal illness and a coincident collapse in funding to resurrect her research, and eventually being awarded the L'Oreal Prize for women in science. She is now head of Comparative Genomics at the Australian National University in Canberra and Director of the Australian Research Council Centre of Excellence for Kangaroo Genomics. I was able to interview her during a visit to her daughter, who lives in the San Francisco Bay area. It was a sunny November afternoon in an outdoor café on the island of Alameda, and over a plate of mussels, some wine ….

**Gitschier:** So much of your work has revolved around your interest in X chromosome inactivation. You've worked on methylation and transcription of X-linked genes in mammalian cells. What is the story in marsupials?

**Graves:** It turns out that in marsupials, DNA methylation doesn't seem to be important! Marsupials are always a shock. Just when you think you know something, you do the same experiments in marsupials and you get the opposite result.

Now we have a genome sequence [from opossum and kangaroo], so we know what genes are on the X and we can just go down the chromosome and ask— are *you* active, are *you* active?

So this is sort of the end—40 years later—of a very long dream of trying to find out how X inactivation evolved.

**Gitschier:** It's probably still not the end!

**Graves:** I was just thinking I shouldn't have said “end.” But X-inactivation is what excites my curiosity more than anything.

**Gitschier:** And that's how you started out, too.

**Graves:** Yes. After that, I went to Berkeley to do my PhD and I had no intention of working on marsupials ever again.

When I came back to Australia, my friend Des Cooper said, “Why don't you map some genes in kangaroos?” I wasn't interested in kangaroos at all. But just to be nice, I did that, and it turned out to be terribly interesting, because the first three genes I looked at were on the X chromosome, like we thought, which was the first indication that the X chromosomes were monophyletic—that they share a common ancestor.

But the next few we looked at weren't! Which was a big shock. It turns out that all the genes on the short arm of the human X are on one place on an autosome in kangaroo. What on earth does that mean? That immediately told us that the human X had an ancient bit and a recent bit.

And that was interesting because it solved a lot of problems. The human X is really strange because the top bit doesn't act like an X at all. It's full of genes that aren't inactivated, and that's because they haven't been on the X for very long.

So that told me something that's been with me my whole life long, which is that sometimes, when you ask a functional question, you get an evolutionary answer. And this has become a guiding principle. Sometimes things are the way they are not because they work better, but because that's the way they evolved. And you can guess function by asking a gene where it has been in the last 100 million years.

**Gitschier:** It must be very thrilling to take a look at something that has been around for a hundred million years and make that observation for the first time.

**Graves:** Absolutely. I started out in molecular genetics and was greatly in awe of the evolutionary biologists who seemed to have their heads in the clouds. I didn't think we even belonged on the same planet! But it never dawned on me how relevant evolutionary thinking was. I didn't realize how all of the answers come from evolution. It's been a real thrill to plug my work into a much bigger framework of how genomes evolved.

**Gitschier:** What do you make of the fact that monotremes exist only in Australia?

**Graves:** They probably didn't. There is a record of one tooth—one monotreme tooth in South America. It is the most amazing story—I just don't know if I believe it or not. To begin with, monotremes just don't have teeth!

But the ancient monotremes *did* have teeth and then they lost their teeth and now have grinding pads instead. But they do have a hatching tooth, so it is still possible to compare with the fossils.

The story I heard was that somebody sat on a tooth and said, “Ouch, what is this? A monotreme tooth!” Well, how on earth would even a palaeontologist know a monotreme tooth? Apparently they are very, very distinctive. And it is pretty much accepted that there were monotremes in South America. Whether they evolved in South America or Australia or what is now Antarctica we don't know. But obviously they spread into Australia and New Guinea, which also has a number of species of *Echidna*. But the platypus is unique to Australia. There were none in New Zealand. But there is this one tooth in Argentina!

**Gitschier:** Now for a silly question: What's it like to hug a koala?

**Graves:** Very uncomfortable. I still have the scars from Bonnie the koala from some photo shoot. I've hugged many koalas, but Bonnie put her arm around me and went *ngghh*. They are not nearly as cuddly as they look.

**Gitschier:** Are there any other marsupials that you've had an intimate relationship with?

**Graves:** I don't do a lot of animal work. I've certainly held a lot of young kangaroos. The little ones are very docile and incredibly cute. But most marsupials are not very good pet material.

**Gitschier:** So none of them have been domesticated.

**Graves:** No, but there is a lot of interest in using marsupials more, particularly kangaroos in the meat trade, because Australia is drying out and sheep and cattle are terribly damaging to the fragile soil.

**Gitschier:** How are the monotremes doing—are they endangered?

**Graves:** We call them “vulnerable”, but that's because the platypus is largely aquatic and it is terribly sensitive to the quality of the water. But the farm runoff has been fixed, and in areas around Melbourne where I live the platypus is coming back, even in suburban Melbourne.

**Gitschier:** Can you see them in the wild? I had to look very carefully even in the aquarium at the Sydney zoo. They are much smaller than I had realized.

**Graves:** It is difficult. I've been on many muddy riverbanks on very cold mornings. There are some places that I can almost always show them to visitors. It's a big thrill even if you don't see too much: a hump in the water and two little eyes looking at you.

It's very difficult to breed the platypus in captivity. One bred in 1934 and then nobody could get any to do it again for another 70 years until they got a new platypusary, with lots of room with long tunnels. Seems to be this one female platypus that has given birth three times. She lays two eggs but only one hatchling survives usually. And she stays with them for 3 months. She makes milk—very complex milk, but she has no teats—it's just exuded from the skin on the abdomen. The young just lie on the skin on the abdomen and lick the milk from her fur.

Lactation is very ancient, but the mammary gland is just a glorified sweat gland. And the mother just lies there for months without getting in food, so she has to be in very good shape.

It was really easy to convince the NIH to sequence the platypus genome because it is so unique—and it is the link between mammals and reptiles. There are so many things about the platypus that are reptilian. It is a mammal—it has fur and it makes milk—but it also lays eggs, and it has a very different structure of the embryo—much more like a reptile.

And to our amazement, the sex chromosomes are more like birds'. When we looked at the sex chromosomes, we found it has ten! Five X and five Y chromosomes. Now we know what genes are on them—they have no homology to human X chromosome, but rather have homology to the bird Z chromosome!

**Gitschier:** And the echidna?

**Graves:** It also has multiple sex chromosomes that are similar to bird.

**Gitschier:** So you mean, to be male you have to have five X's and five Y's. And they are completely different from each other?

**Graves:** Absolutely!

**Gitschier:** How do they do that?

**Graves:** XY pairs have a little bit of homology. A little bit of X1 has homology to Y1 and then Y1 at the other end has a little bit of homology to X2 and then the other end of X2 is homologous to Y2. So these pseudoautosomal regions pair, and at meiosis you can actually see ten chromosomes in a chain—XYXYXY, etc. How they segregate is a mystery—we've never actually caught them at anaphase, but we think all the X's must go one way and all the Y's the other, because we've never actually seen a sperm or spermatocyte with both X's and Y's in them.

But we're not so surprised because there are multiple chromosomes in some plants like the evening primrose, and in some spiders. It seems to have happened when two different chromosomes swap bits and must pair in a chain of four, and then one of these swaps bits, and so on. Functionally quite crazy, but once it happens, it is stuck, and must make the best of it.

It really amused me to be told once that our *Nature* paper on platypus sex chromosomes was featured on the “Discovery” Web site. And I said, “Oh, that's wonderful,” and they said, “Well maybe you don't know that the Discovery Web site is creationist, and your paper is put on there as an example of intelligent design!”

I said, “That's the dumbest thing I've ever heard!” And that was the inspiration for my “dumb design” Web site, which I'm setting up now with my L'Oreal prize money, as examples of how evolution can explain things very simply that seem to make no functional sense at all. So that's going to be my first example, of something that happened once, accidentally, that now can't un-happen, but how systems work around these accidents to make the best of a bad job.

It truly distresses me to see kids being brought up to believe in utter nonsense [creationism/intelligent design].

**Gitschier:** Is that true in Australia, too?

**Graves:** Not as true in Australia as in the US. But there is a lot of pressure to accept the teaching of utter nonsense in school. I think we are raising a credulous generation who will believe anything as long as they read it in *Reader*'*s Digest*. It's so dangerous to encourage people to believe what they are told rather than what they observe.

Over many years I've been distressed to find that our students come to us without the ability to observe anything! Our students will sit in front of microscopes and draw things that aren't there! And I'll say, “Well, that's very nice to draw chromosomes with a spindle attached. Do you see a spindle attached?”

“Oh yes!”

Of course, you can't *see* spindles! So I look down the microscope and say, “I don't see a spindle.” And they say, “But I know it is there, because my textbook has a spindle.”

As long as you are drawing things that aren't there because somebody tells you they are there, you are in deep, deep trouble.

And I think it is much deeper than just believing or not believing in evolution, you've got to change education to encourage children to go looking for themselves. And start thinking to themselves: well—who's right?—what I see or what someone tells me?

So I'm becoming very interested in education, particularly of young children, which is where I think the rot sets in. Science is not taught well even at high school level, and at primary school level it is taught by people who are generally scared of science! Anybody who has anything to do with kids this age knows they are incredibly observant and incredibly clever at working out how what they observe relates to other things. Somehow that just gets lost. I'd love to see more attention on encouraging young kids to make their own hypotheses—crazy though they may be.

**Gitschier:** Let's turn to the testes-determining factor and the race to clone the gene. I'd love to hear your recall of that period.

**Graves:** I'll tell you the story as it happened because it is a good yarn.

I had no pretensions of working on sex-determination at all. But of course I was interested, and I was watching this *war* going on between David Page's group in Boston and Peter Goodfellow's group in London regarding finding the testes-determining factor.

When David Page's paper on ZFY [the putative testes-determining factor] came out in *Cell* in 1987 I thought, “Wow, this is gorgeous, beautifully done.” I didn't think we would have anything to contribute. But David then called me up that same night. I didn't know him at all. He said they would really like to show this gene is on the Y chromosome in marsupials. So he sent us the probe.

Oddly enough, Peter Goodfellow sent us an independent probe of the same gene. He just sent the probe, he didn't even ask in advance!

My student Andrew Sinclair—it was the last week of his PhD work—had looked at other genes on the short arm of the X chromosome in humans and had shown that they are autosomal in marsupials.

The gene David sent us [which came from the Y chromosome] also had a friend [a homolog] in that short arm of the human X chromosome. We thought: that's interesting, wonder where ZFY will be in marsupials.

Well, Andrew called me up late one night and said, hope you're sitting down because ZFY is not on the Y chromosome: it is on Chromosome 5—which is a very funny place to keep your sex-determining gene.

So I said, “Don't be silly, look at more cells.” This is the old days where we used radioactive in situ hybridization and had to count silver grains over hundreds of cells. The next morning he had absolutely incontrovertible evidence that it was in the same patch of autosomal genes that we had shown should have been on the X, but weren't.

So I told David, and of course he didn't believe it, because that meant that ZFY was not the testes-determining factor. He thought there was just something very strange about marsupials.

In the meantime, we got the same result with the probe Peter had sent us, and he was *very* keen to publish [it became a cover article in *Nature*]. Andrew later went to Peter Goodfellow's lab, which had been organized before all this had happened, and it was he who cloned the SRY gene, which was the true testis-determining gene.

I was right in the middle of the war zone. For an innocent little Australian, that was quite a wake-up call! But I've become good friends with both of them.

**Gitschier:** About this period when you lost a lot of funding and had your illness—I'd like to talk to you about that because it may be inspirational to others of us when we go through troughs.

**Graves:** It was strange. My lab was sailing along. We had our cover story in *Nature*, and another paper in *Nature*. I didn't have a care in the world. Then, I failed to renew two grants. Maybe I was just too cocky.

When a week later I collapsed from a brain bleed, the rumor went around that “Jenny's died from a broken heart!” Everything seemed to be conspiring to say, “That's the end of you!”

But I was fortunate. I had a great neurosurgeon and a wonderful collaboration with Art Riggs in Los Angeles on the platypus, and he funded a technician in my lab. I called him from my hospital bed, telling him I was in dire trouble and asking whether he could support her for another year. That absolutely saved me—to keep that expertise in the lab! And the university gave a scholarship to another technician in my lab, so I could continue. And I did have two loyal graduate students.

This was 1992–1993. Arterio–ventricular malformation, a congenital thing, which is not all that uncommon, but it was in a very bad place—the fourth ventricle. The neurosurgeons told me it would take me 18 months to get back on my feet, which was true. I couldn't see, I couldn't walk, and I was being sick all the time.

I had a lot of time to think, and I could type! I had a proofreader. I wrote five grants and I got the lot. From three people in the lab, I had 18 the next year.

**Gitschier:** During that period, though, did you consider doing something completely different?

**Graves:** People said, “Now is the chance to really think about your life and what you want to do.” I did—and I thought, “Yes, I want more of the same!”

I did think about other things I could do, particularly if my vision was to be impaired, but I very quickly decided I love what I'm doing and couldn't wait to get back to the lab, and I signed two contracts for books, one of which I'm still working on—the molecular biology of sex chromosomes. I had so much fun. I found I knew so much I could just write it without referencing anything, and then when I recovered, I started plugging in real data and references.

**Gitschier:** So you may not retire.

**Graves:** I will retire from writing grants, I swear. I have enough grant money till 2010. But I'll be 69 by that time and I'm not sure I want to keep running a wet lab. It would be a good time to return to Melbourne, where I live, and I'll continue writing. I think I'll be as happy as a lark and co-supervise students at the Uni [University of Melbourne]. I want to learn to be a bioinformatics person. The world is full of data.

**Gitschier:** When did it occur to you that studying marsupials and monotremes would help to solve such important questions about evolution?

**Graves:** I'm ashamed to admit it didn't dawn on me right away. I found it fascinating to map genes in marsupials, but it still didn't get to me that this was *important.*

The beginning of that realization was when Steve O'Brien came to Australia in 1984. He saw right away that animals distantly related to human would be extremely powerful. So it was really he who convinced me to believe in what I was doing. And once I had started on that track, it was easy to find more and more things that one could look at. Anything you like can be looked at through the spectrum of evolution, and if you have systems that are so divergent, comparisons are extremely powerful. And of course, that was before sequencing, we were just looking at arrangements of genes, simple mapping. But already it was obvious that the out-group gave you real power to tell you how the genome was rearranged.

**Gitschier:** You mean monotremes.

**Graves:** Monotremes are an out-group to marsupials and placental mammals, marsupials are an out-group to placental mammals. Chicken is the out-group to all the mammals.

All of a sudden I realized I was sitting on a gold mine. And I felt such a sense of responsibility. Here is a very Australian gold mine, and I've got to get out and beat the hedges and tell people what we've got in Australia. Because Australians, curiously, don't realize what we could be doing with our own native flora and fauna. It is not a well-funded field.

**Gitschier:** So, in a sense, moving back to Australia was the best career move you could have ever made.

**Graves:** It was! I didn't think of it that way. I never expected I could be doing something unique and important so far away from the action.

I always tell young Australians, knowing there are such huge resources in the north [Northern Hemisphere] that we are competing with, if you can find something unique, then you know what you are producing is unique. Then you just have to worry whether it is unique and *boring* or unique and exciting. And of course, you don't know, sometimes you have to do some work to find out. You can pretty soon see whether you are getting general principles out of it.

We were very lucky. The whole ZFY business really showed me that we were on the right track.

I used to give talks on kangaroo genomes in the US and people would laugh! If I showed a koala picture they would say “Ahhhhh”, and if I'd put up a platypus they'd fall off their chairs laughing. I thought, “At least they're paying attention.”

After the SRY story, nobody was laughing at kangaroos any more.

